# Data-Driven Path Analytic Modeling to Understand Underlying Mechanisms in COVID-19 Survivors Suffering from Long-Term Post-COVID Pain: A Spanish Cohort Study

**DOI:** 10.3390/pathogens11111336

**Published:** 2022-11-12

**Authors:** César Fernández-de-las-Peñas, Bernard X. W. Liew, Manuel Herrero-Montes, Pablo del-Valle-Loarte, Rafael Rodríguez-Rosado, Diego Ferrer-Pargada, Randy Neblett, Paula Paras-Bravo

**Affiliations:** 1 Department of Physical Therapy, Occupational Therapy, Physical Medicine and Rehabilitation, Universidad Rey Juan Carlos (URJC), 28922 Alcorcón, Spain; 2 School of Sport, Rehabilitation and Exercise Sciences, University of Essex, Colchester CO4 3SQ, UK; 3 Departamento de Enfermería, Universidad de Cantabria, 39008 Santander, Spain; 4 Grupo de Investigación en Enfermería, Instituto de Investigación Sanitaria Valdecilla (IDIVAL), 39011 Santander, Spain; 5 Department of Internal Medicine, Hospital Universitario Severo Ochoa, 28911 Leganes, Spain; 6 Servicio de Neumología, Hospital Universitario Marqués de Valdecilla, 39008 Cantabria, Spain; 7 PRIDE Research Foundation, Dallas, TX 75235, USA

**Keywords:** pain, COVID-19, post-COVID, bayesian network, structural equation modeling

## Abstract

Pain can be present in up to 50% of people with post-COVID-19 condition. Understanding the complexity of post-COVID pain can help with better phenotyping of this post-COVID symptom. The aim of this study is to describe the complex associations between sensory-related, psychological, and cognitive variables in previously hospitalized COVID-19 survivors with post-COVID pain, recruited from three hospitals in Madrid (Spain) by using data-driven path analytic modeling. Demographic (i.e., age, height, and weight), sensory-related (intensity or duration of pain, central sensitization-associated symptoms, and neuropathic pain features), psychological (anxiety and depressive levels, and sleep quality), and cognitive (catastrophizing and kinesiophobia) variables were collected in a sample of 149 subjects with post-COVID pain. A Bayesian network was used for structural learning, and the structural model was fitted using structural equation modeling (SEM). The SEM model fit was excellent: RMSEA < 0.001, CFI = 1.000, SRMR = 0.063, and NNFI = 1.008. The only significant predictor of post-COVID pain was the level of depressive symptoms (β=0.241, *p* = 0.001). Higher levels of anxiety were associated with greater central sensitization-associated symptoms by a magnitude of β=0.406 (*p* = 0.008). Males reported less severe neuropathic pain symptoms (−1.50 SD S-LANSS score, *p* < 0.001) than females. A higher level of depressive symptoms was associated with worse sleep quality (β=0.406, *p* < 0.001), and greater levels of catastrophizing (β=0.345, *p* < 0.001). This study presents a model for post-COVID pain where psychological factors were related to central sensitization-associated symptoms and sleep quality. Further, maladaptive cognitions, such as catastrophizing, were also associated with depression. Finally, females reported more neuropathic pain features than males. Our data-driven model could be leveraged in clinical trials investigating treatment approaches in COVID-19 survivors with post-COVID pain and can represent a first step for the development of a theoretical/conceptual framework for post-COVID pain.

## 1. Introduction

After the acute phase of severe acute respiratory syndrome coronavirus-2 (SARS-CoV-2) infection, some individuals report continued problems with a variety of symptoms, with fatigue and dyspnoea being the most prevalent, e.g., post-COVID-19 condition [1]. The World Health Organization has recently proposed the following definition: “Post-COVID-19 condition occurs in people with a history of probable or confirmed SARS-CoV-2 infection, usually 3 months from the onset of COVID-19 with symptoms that last for at least 2 months and cannot be explained by an alternative diagnosis. Common symptoms include fatigue, shortness of breath, and cognitive dysfunction (but also others), and generally have an impact on everyday functioning. Symptoms might be new-onset after initial recovery from an acute COVID-19 episode or persist from the initial illness. Symptoms might also fluctuate or relapse over time” [2]. Pain is a highly prevalent post-COVID symptom, which is present in up to 50% of patients with post-COVID-19 condition [3,4,5].

The phenotyping of post-COVID pain could increase our current understanding of potential mechanisms and also orientate personalized treatment following mechanism-based classifications. Different features of post-COVID pain have been identified in some studies. First, a cohort study identified that post-COVID pain exhibits musculoskeletal pain features in 45% of patients at eight [6] and twelve [7] months after hospitalization. These studies observed that almost 20% of patients reported widespread pain symptoms [6,7]. Widespread pain symptomatology is associated with the presence of sensitization mechanisms. In fact, preliminary evidence suggests the presence of sensitization in individuals with post-COVID pain [8]. Sensitization is the underlying concept of nociplastic pain, defined as “pain that arises from altered nociception without clear evidence of actual or threatened tissue damage causing the activation of peripheral nociceptors or evidence for disease or lesion of the somatosensory system causing pain” [9]. Nociplastic pain conditions are associated with exaggerated pain responses but also with other central nervous system-derived symptoms such as fatigue, sleep problems, memory loss, and psychological disturbances [10]. All these central nervous system-derived symptoms have been observed in subjects with post-COVID-19 condition [11,12]. Additionally, other studies have also observed that some patients with post-COVID pain exhibit neuropathic pain features [13,14].

The mechanisms underpinning the pain reported by patients with post-COVID-19 condition is currently unclear and could involve complex interactions between biological and cognitive/behavioral factors. Complex interactions between multiple variables, where variables can simultaneously be a predictor, mediator, and outcome, lend themselves to analysis using structural equation modeling (SEM) [15]. SEM requires the specification of a structural model which determines how variables influence each other. The structure of the model is typically determined using a combination of empirical research, substantive theory, and clinical expertise. Another method of determining the model’s structure is using a data-driven structural learning approach, such as a Bayesian network (BN) [16]. A data-driven structural learning approach can be especially useful when substantive theory and existing research are not conclusive, such as with the mechanisms in post-COVID pain. Network methodologies, such as BN analyses, could help to better explain the complexity of post-COVID pain. The learned structural model using a BN can then be fitted using traditional SEM analysis for inferential statistics.

No previous study has used BN analysis for phenotyping post-COVID pain. The primary aim of this study is to understand the multivariate and complex interactions between clinical, sensory-related, psychological, and cognitive variables in previously hospitalized COVID-19 survivors with post-COVID pain by using data-driven path analytic modeling.

## 2. Methods

### 2.1. Participants

We included a cohort of subjects who were previously hospitalized because of SARS-CoV-2 infection during the first wave of the pandemic (March–April 2020) in three hospitals in Spain. The diagnosis of SARS-CoV-2 infection was determined with a real-time reverse transcription-polymerase chain reaction (RT-PCR) assay of nasopharyngeal and/or oral swab samples and the presence of clinical/radiological findings at hospital admission. Patients were invited to participate in this study if: 1. they reported lingering pain symptoms for at least three months after hospital discharge, post-SARS-CoV-2 infection, and 2. the pain could not be attributed to any other underlying medical condition (e.g., arthritis). Participants were excluded if they reported a history of pain before the infection or any existing medical comorbidity that could explain the pain symptoms. This study was approved by all the Institutional Ethics Committees of the involved hospitals (INDIVAL Cantabria 2020.416; URJC0907202015920; HUIL/092-20, HUFA 20/126; HSO 25112020).

### 2.2. Data Collection

Participants were scheduled for a face-to-face interview at a follow-up period longer than one year after hospitalization. A structured data collection questionnaire including clinical data of their symptoms and several patient-reported outcome measures (PROMs) was used. Age, weight, height, intensity (numerical pain rating scale, NPRS: 0–10 points), and duration of pain symptoms were collected as demographic and clinical variables, respectively. The PROMs evaluated sensory-related (e.g., sensitization-associated symptoms and neuropathic pain features), psychological (anxiety levels, depressive levels, and sleep quality), and cognitive (catastrophism and kinesiophobia) aspects.

### 2.3. Sensory-Related Variables

The presence of central sensitization-associated symptomatology was assessed with the Central Sensitization Inventory (CSI, total score 0–100 points) [17]. Total CSI scores have demonstrated good psychometric properties for assessing sensitization-associated symptoms in people with chronic pain [18]. A total score >40 points has been recommended as a cutoff to indicate that symptoms may be associated with central sensitization [19].

The presence of neuropathic pain symptoms was assessed with the self-report Leeds Assessment of Neuropathic Symptoms (S-LANSS, scored 0–24 points) [20] and PainDETECT (scored 0–30 points) [21]. According to the S-LANSS, a score ≥12 points suggests the presence of pain of neuropathic origin [20], and according to the PainDETECT, a score of >18 points suggests the likely presence of neuropathic features [21].

### 2.4. Psychological Variables

The Hospital Anxiety and Depression Scale (HADS) was used to evaluate the presence of anxiety symptoms (HADS-A, 7 items, scored 0–21 points) and depressive symptoms (HADS-D, 7 items, scored 0–21 points) [22].

The Pittsburgh Sleep Quality Index (PSQI, 24 items, scored 0–21 points) was used to assess the quality of sleep, usual bed-time, usual wake time, the number of actual hours slept, and the number of minutes to fall asleep [23].

### 2.5. Cognitive Variables

Pain catastrophizing was assessed with the Pain Catastrophizing Scale (PCS, 13 items, scored 0–52 points) [24]. The PCS evaluates specific aspects of catastrophizing, including rumination, magnification, and feelings of despair concerning one’s pain experience.

Kinesiophobia, i.e., fear of movement, was assessed with the 11-item Tampa Scale Kinesiophobia (TSK-11, scored 11–44 points) [25]. Kinesiophobia symptoms are considered to be minimal when the TSK-11 score ≤22 points, low from 23 to 28 points, moderate from 29 to 35 points, and high ≥36 [26].

### 2.6. Statistical Analysis

#### 2.6.1. Packages

All analyses were performed using the R software (v4.1.2, R Core Team, Vienna, Austria). The following packages were used: mice [27] for data imputation, lavaan [28] for SEM analysis, semPlot [29] for visualizing SEM paths, and bnlearn [30] for BN structural learning.

#### 2.6.2. Missing Data Management

The proportion of missing data was less than 5%. Multiple imputations were performed on all variables with missing values using the multivariate imputation by chained equations method [27]. The random forest method was used for data imputation. We generated 20 imputed datasets using a maximum number of iterations of 30 for each imputation. For subsequent analysis, we only used a single imputed dataset. Prior to BN and SEM analysis, all continuous variables were scaled to have a mean of 0 and standard deviation of 1.

#### 2.6.3. Bayesian Network (BN)

BN is a graphical modeling technique [31] that can leverage either data alone or data combined with an expert’s prior knowledge to learn multivariate pathway models. BN modeling involves two stages: (1) structure learning, identifying which arcs are present in the graphical model, and (2) parameter learning, estimating the parameters that regulate the strength and the sign of the relationships. In the current study, BN was used for structural learning only and the learned parameters were not used.

BN can easily include prior knowledge during the model-building process. In the BN framework, prior knowledge of known relationships can be included in the model as blacklist and whitelist arcs. Blacklisted arcs are always excluded from the model’s structure, whilst whitelisted arcs are always included in the structure. Blacklist arcs are those that contravene known biological or physical mechanisms. In the current study, we imposed the following blacklist that no arcs point to the variables of BMI and Sex, since no variable can affect them.

We made use of model averaging to reduce the potential of including spurious relationships in the BN using bootstrap resampling (B = 1000). The original data were resampled randomly with replacement 1000 times and for each resampled data, structure learning was performed using the hill-climbing (HC) algorithm. An “average” consensus model was calculated by selecting those arcs that had a frequency greater than 70% in the bootstrapped samples to create a sparse and interpretable network [32]. This DAG was again used for SEM analysis.

#### 2.6.4. Structural Equation Modeling (SEM)

**Figure 1:**
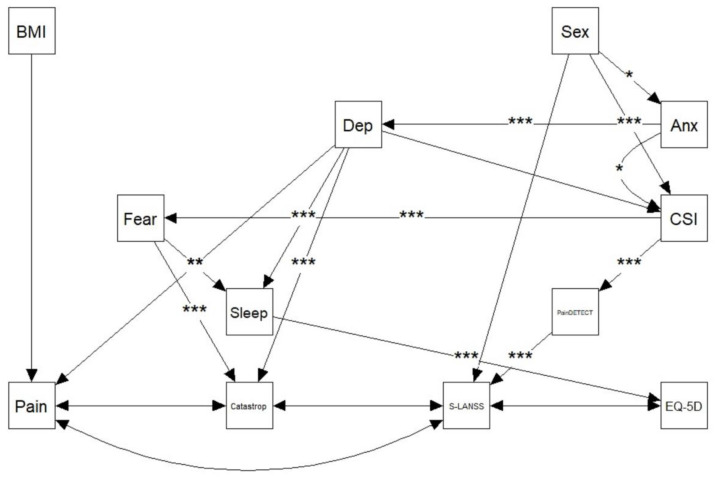
Network structure learned from Bayesian network, with arc parameters learned from structural equations modeling. * *p* < 0.05, ** *p* < 0.01, *** *p* < 0.001. Abbreviations. BMI: Body mass index; PCS: Pain Catastrophizing Scale; S-LANSS: self-administered Leeds Assessment of Neuropathic Symptoms and Signs; EQ-5D-5L: European Quality of Life Five Dimension; CSI: Central Sensitization Inventory; HADS-A: Hospital Anxiety and Depression Scale (Anxiety Scale); HADS-D: Hospital Anxiety and Depression Scale (Depression Scale); PSQI: Pittsburgh Sleeping Quality Index; TSK: Tampa Scale for Kinesiophobia. → indicates a regression path; ↔ indicates co-variance.

We used the network structure determined from the BN for SEM analysis (Figure 1). Maximum likelihood was used to estimate the model’s parameters, whilst the Bollen–Stine bootstrap was used to quantify the standard error and probability value of the test statistic. An excellent SEM model fit is determined when two of the four fit indices exceed the thresholds: a root-mean-square error of approximation (RMSEA) ≤0.05; standard root mean residual (SRMR) ≤0.05; confirmatory fit index (CFI) ≥0.95; non-normed fit index (NNFI) ≥0.95) [33]. For the estimated parameters, a *p*-value < 0.05 was considered to be statistically significant.

We calculated the indirect mediation effects of the following hypothesized associations based on a clinical model: (1) depressive levels on anxiety/central sensitization-associated symptoms (CSI) association; (2) central sensitization-associated symptoms (CSI) on sex/neuropathic pain features (S-LANSS) association; (3) fear (kinesiophobia, TSK-11) on depressive levels/pain catastrophism association; (4) the combined serial effects of central sensitization-associated symptoms (CSI) and fear (kinesiophobia, TSK-11) on the depressive levels/sleep quality relationship, by using the product of coefficients approach [34].

## 3. Results

Of 200 previously hospitalized COVID-19 survivors screened for participation, 146 participants (54.5% female, age: 57.3 ± 11.7 years old) were included and analyzed, based on the inclusion/exclusion criteria. Forty-four participants were mainly excluded because they did not report the presence of pain in their post-COVID symptoms. The included participants were assessed at a follow-up period of 18.8 ± 1.8 months after hospital discharge. Table 1 summarizes the descriptive features of the cohort. Fit for the SEM model was excellent (RMSEA < 0.001, CFI = 1.000, SRMR = 0.063, and NNFI = 1.008) (Figure 1). The model and associated standardized regression weights, standard errors, 95% confidence intervals (CI), and *p*-values can be found in Table 2.

The only significant predictor of post-COVID pain intensity was depressive levels, where a 1 SD increase in depressive symptoms resulted in a 0.241 SD increase in pain intensity (Table 2, Figure 1). A greater level of anxiety was associated with a greater level of central sensitization-associated symptoms (CSI) by a magnitude of β=0.406 (*p* = 0.007, Table 2, Figure 1). Males exhibited a −1.50 SD units (*p* < 0.001) lower S-LANSS score than females (Table 2, Figure 1). A greater level of depression resulted in worse sleep quality (β=0.298, *p* < 0.001) and greater catastrophizing levels (β=0.345, *p* < 0.001, Table 2, Figure 1). Of the four indirect effects that were tested, the only significant indirect pathway was the sex-influenced S-LANSS via the serial effects of CSI and PainDETECT (β=0.063, *p* = 0.001, Table 2, Figure 1). Several of the indirect effects determined from the BN were not significant, such as the effect of depressive levels on anxiety/central sensitization-associated symptom pathway (β=0.089, *p* = 0.397); the serial effect of central sensitization-associated symptoms and fear (kinesiophobia) on the depression/sleep quality relationship (β=0.011, *p* = 0.436); the serial effect of central sensitization-associated symptoms and neuropathic pain on the sex and S-LANSS (β=0.027, *p* = 0.426, Table 2, Figure 1).

## 4. Discussion

An interaction among biological, cognitive, and emotional factors is thought to be related to the pathogenesis of post-COVID pain, which suggests suitability for analysis within the SEM framework. This study applied BN to create a data-driven model to better understand the complex interactions between clinical, sensory-related, psychological, and cognitive variables in previously hospitalized COVID-19 survivors. We discuss here the most relevant associations confirmed or refuted by the observed model.

### 4.1. Emotional Aspects and Post-COVID Pain

The fear-avoidance model posits that maladaptive cognitions, e.g., pain catastrophizing and kinesiophobia (fear of movement), and psychological distress are drivers of pain intensity and related disability [35]. A recent meta-analysis supported the fear-avoidance model by showing significant associations between kinesiophobia, catastrophizing, and pain vigilance with psychological distress, pain intensity, and pain-related disability [36]. Our data-driven model found that depressive symptoms were the only variable associated with the intensity of post-COVID pain. The association between pain and depressive symptoms is well described in the literature. A bidirectional effect is suggested, in which pain can lead to increased depressive symptoms and depressive symptoms can lead to increased pain. Our data-driven model also revealed that a greater level of depression was associated with worse sleep quality. The meta-analysis conducted by Alimoradi et al. found that sleep problems were associated with higher levels of depression during the COVID-19 outbreak [37]; again, the association can be bidirectional: poor sleep can result in daytime sleepiness, low energy, and depressed mood, and higher levels of depressive symptoms can lead to worse quality of sleep, creating a vicious cycle.

Higher levels of depressive symptoms were also associated with greater levels of pain catastrophizing. Pain catastrophizing refers to maladaptive cognitions that are associated with pain-related fear and emotional distress. The association between maladaptive catastrophizing cognitions, emotional distress, and depressive symptoms can provoke a vicious cycle, which can help perpetuate pain.

Another maladaptive response to pain that can promote pain chronification is fear of motion, i.e., kinesiophobia. Strong evidence supports that higher levels of kinesiophobia are associated with higher pain intensity and more related-disability in people with chronic pain of musculoskeletal origin [38]. Interestingly, the role of kinesiophobia in our data-driven model was small, since this variable was only significantly associated with sleep quality and pain catastrophizing, without any potential serial effect.

### 4.2. Central Sensitization-Associated Symptoms and Post-COVID Pain

Central sensitization is clinically characterized by widespread or multisite allodynia or hyperalgesia, which cannot be entirely explained by nociceptive or neuropathic mechanisms, and is often associated with a range of other associated symptoms, including fatigue, sleep problems, cognitive/emotional disturbance, and multisensory hypersensitivity [39]. Though it often develops following injury and tissue damage, central sensitization-related pain and associated symptoms can occur without any injury process in prone individuals, with perhaps a genetic predisposition [40]. It has become increasingly recognized that central sensitization is likely an underlying mechanism across many chronic pain conditions [41]. The hypothesis of sensitization mechanisms in post-COVID pain has been previously formulated by Goudman et al. [8]. Our data-driven model found that greater levels of anxiety directly resulted in higher central sensitization-associated symptomatology as assessed with the CSI. An association between anxiety/emotional distress and central sensitization-associated symptoms supports the findings of previous studies in individuals with chronic pain [42,43]. Anxiety and related cognitive/emotional symptoms can negatively impact the central nervous system by reducing descending pain inhibition and amplifying pain-related signals [44], resulting in pain hypersensitivity.

### 4.3. Sex and Post-COVID Pain

Previous studies have found that the female sex is a risk factor for developing post-COVID symptoms, including post-COVID pain [45]. Sex differences in other chronic pain conditions, such as fibromyalgia, have also been found with a higher prevalence of females to males [46]. The data-driven model in our study revealed that the presence of neuropathic pain features, assessed with the S-LANSS, was higher in females than in males. Current hypotheses include sex differences in the molecular response, i.e., a sexually dimorphic response of pain signaling, particularly in the microglia against nociceptive stimuli [47]; sex differences in pain perception [48] and cognitive/emotional processing [49] have also been hypothesized.

Our data-driven model saw that sensitization-associated symptoms were associated with neuropathic features, assessed with the PainDETECT. This association supports that a neuropathic pain component plays a relevant role in the phenotype of post-COVID pain. In fact, the prevalence of neuropathic pain features in subjects with post-COVID pain is 20–25% [13]. Nevertheless, an important finding identified by our BN model was that the association between sensitization-associated symptoms (i.e., CSI) and neuropathic features (i.e., PainDETECT and S-LANSS) was indirectly mediated by sex. This interaction supports that sex is a risk factor for the development of neuropathic features in people with post-COVID pain and mediating the presence of sensitization-associated symptoms.

### 4.4. Clinical Application

The results of this study have several clinical implications. Based on the presence of central nervous system symptoms and a lack of objective pain generators, the pain experienced by some COVID-19 survivors could be classified as “nociplastic pain” [9]. The BN in the present study revealed a complex model of interactions between psychological and biological variables. Accordingly, this data-driven analysis suggests that post-COVID pain represents a multidimensional condition, and therefore, multimodal approaches are recommended. For instance, since emotional stress (e.g., depression and anxiety) and maladaptive cognitions (e.g., pain catastrophizing) are modifiable risk factors of chronic pain, treatment of these symptoms should be considered in the management of post-COVID-related pain. Clinicians should consider individually tailored multimodal treatments combining neuroscience pain education, physical therapy/exercise, stress management training, and appropriate medication management in this population.

In fact, considering post-COVID pain as a “nociplastic condition” should lead to clinical and patient-to-patient adaptation when programming exercises [50] in people with post-COVID-19 condition [51]. Accordingly, the associations observed in our data-driven model should be individually identified for each patient with post-COVID pain for the prescription of personalized exercise and complementary interventions. Therefore, these findings suggest that the management of post-COVID pain should include multimodal therapeutic approaches targeting emotional and cognitive aspects (i.e., cognitive behavior and coping strategies), central sensitization-associated mechanisms (i.e., neuro-modulatory pain approaches, e.g., exercise or pain education), as well as advice on healthy lifestyles (e.g., nutrition or sleep management). These interventions should be adapted to the clinical presentation of each individual since the influence of each of the identified variables in the current study will be unique.

### 4.5. Strengths and Limitations

This study is not without limitations. First, the cross-sectional nature of the design precludes the ability to disentangle between-subjects from within-subject relationships. Accordingly, although we applied a BN, due to the cross-sectional nature of the design, a causal relationship should be considered with caution. A future longitudinal analysis will enable the separation of between-and within-subject pathways. Additionally, we should recognize that the sample size could be considered relatively small. Based on a rule-of-thumb used frequently in SEM, with 10 observations required for 1 modeled variable [52], with 12 variables included in the model, it requires 120 observations. Second, we should not exclude a potential context bias. This study was conducted in a European country highly affected by the COVID-19 outbreak, Spain, and accordingly, cultural characteristics could be also involved in pain perception in patients with post-COVID-19 condition. Third, current data can be only applicable to previously hospitalized COVID-19 survivors. Fourth, we excluded patients with pre-existing pain experienced before the infection since this is a risk factor for developing post-COVID pain [6,7]. We do not know if the presence of pain before the infection would have led to the same model. Accordingly, the results from the current study should be considered exploratory and should be confirmed in future validation studies.

## 5. Conclusions

This study presents a data-driven model for post-COVID pain, which included psychological and emotional factors, sensitization-associated symptoms, and sleep quality. In addition, maladaptive cognitive behaviors such as pain catastrophizing are also associated with emotional aspects such as depressive levels. Finally, sex seems to play a relevant role in neuropathic pain features and sensitization-associated symptomatology. Current data would support that post-COVID pain could be phenotyping as a “nociplastic” pain condition and accordingly, this should lead to clinical and patient-to-patient adaptation when designing treatment approaches. The current data-driven model could be leveraged in clinical trials investigating treatment approaches in COVID-19 survivors with post-COVID pain and can represent a first step for the development of a theoretical/conceptual framework for post-COVID pain.

## Figures and Tables

**Table 1:** Descriptive characteristics of the cohort (n = 146).

Variables	Mean (SD)
Age (years)	57.3 (11.7)
BMI (kg/m^2^)	29.25 (5.2)
Pain duration (months)	18.8 (1.8)
Pain intensity (NPRS, 0–10)	5.6 (1.7)
Anxiety (HADS-A, 0–21)	5.2 (4.2)
Depression (HADS-D, 0–21)	4.9 (4.3)
Sleep (PSQI, 0–21)	8.0 (4.2)
PainDETECT (−1 to 38)	7.0 (6.2)
S-LANSS (0–24)	7.5 (8.5)
CSI (0–100)	33.9 (17.2)
Catastrophizing (PCS, 0–52)	12.3 (12.0)
Fear (TSK-11, 0–44)	24.0 (8.6)
EQ-5D-5L (0–1)	0.8 (0.2)

BMI: Body mass index; PCS: Pain Catastrophizing Scale; S-LANSS: self-administered Leeds Assessment of Neuropathic Symptoms and Signs; EQ-5D-5L: European Quality of Life Five Dimension; CSI: Central Sensitization Inventory; HADS-A: Hospital Anxiety and Depression Scale (Anxiety Scale); HADS-D: Hospital Anxiety and Depression Scale (Depression Scale); PSQI: Pittsburg Sleeping Quality Index; TSK-11: Tampa Scale for Kinesiophobia.

**Table 2:** Standardized parameter estimates.

DV	IV	Coef	SE	Pval	2.5%	97.5%	Type
Pain	BMI	0.015	0.086	0.860	−0.154	0.185	Reg
Pain	Dep	0.241	0.075	0.001	0.095	0.387	Reg
Anx	Sex	0.179	0.079	0.024	0.024	0.334	Reg
Dep	Anx	0.745	0.064	0.000	0.620	0.869	Reg
Sleep	Dep	0.298	0.071	0.000	0.158	0.438	Reg
Sleep	Fear	0.201	0.080	0.012	0.045	0.357	Reg
PainDETECT	CSI	0.406	0.071	0.000	0.267	0.545	Reg
S-LANSS	Sex	−0.150	0.052	0.004	−0.251	−0.049	Reg
S-LANSS	PainDETECT	0.488	0.061	0.000	0.367	0.608	Reg
CSI	Sex	0.319	0.063	0.000	0.196	0.441	Reg
CSI	Anx	0.406	0.153	0.008	0.106	0.706	Reg
CSI	Dep	0.119	0.146	0.415	−0.167	0.405	Reg
Catastrop	Dep	0.345	0.079	0.000	0.191	0.499	Reg
Catastrop	Fear	0.494	0.066	0.000	0.364	0.624	Reg
Fear	CSI	0.458	0.074	0.000	0.313	0.603	Reg
EQ-5D	Sleep	−0.305	0.071	0.000	−0.443	−0.167	Reg
Indirect effect 1 (Anx→Dep→CSI)		0.089	0.105	0.397	−0.116	0.294	Med
Indirect effect 2 (Dep→CSI→Fear→Sleep)		0.011	0.014	0.436	−0.017	0.039	Med
Indirect effect 3 (Sex→CSI→PainDETECT→S-LANSS)		0.063	0.019	0.001	0.026	0.100	Med
Indirect effect 4 (Dep→CSI→Fear→Catastrop)		0.027	0.034	0.426	−0.039	0.093	Med
Pain	Pain	0.942	0.036	0.000	0.871	1.013	vCov
Anx	Anx	0.968	0.028	0.000	0.912	1.024	vCov
Dep	Dep	0.445	0.095	0.000	0.260	0.631	vCov
Sleep	Sleep	0.845	0.051	0.000	0.746	0.945	vCov
PainDETECT	PainDETECT	0.835	0.058	0.000	0.722	0.948	vCov
S-LANSS	S-LANSS	0.764	0.054	0.000	0.658	0.870	vCov
CSI	CSI	0.591	0.056	0.000	0.481	0.701	vCov
Catastrop	Catastrop	0.565	0.055	0.000	0.457	0.672	vCov
Fear	Fear	0.790	0.068	0.000	0.657	0.923	vCov
EQ-5D	EQ-5D	0.907	0.043	0.000	0.823	0.991	vCov
Pain	S-LANSS	−0.051	0.081	0.524	−0.210	0.107	vCov
Pain	Catastrop	−0.145	0.071	0.042	−0.285	−0.005	vCov
Pain	EQ-5D	0.047	0.078	0.544	−0.105	0.199	vCov
S-LANSS	Catastrop	−0.001	0.058	0.991	−0.115	0.113	vCov
S-LANSS	EQ-5D	0.064	0.069	0.349	−0.070	0.199	vCov
Catastrop	EQ-5D	−0.085	0.088	0.332	−0.258	0.087	vCov
BMI	BMI	1.000	0.000		1.000	1.000	vCov
BMI	Sex	0.073	0.000		0.073	0.073	vCov
Sex	Sex	1.000	0.000		1.000	1.000	vCov

Abbreviations. BMI: Body mass index; Catastrop: catastrophizing; S-LANSS: self-administered Leeds Assessment of Neuropathic Symptoms and Signs; EQ-5D: European Quality of Life Five Dimension; CSI: Central Sensitization Inventory; Anx: anxiety; Dep: depressive symptoms; DV: dependent variable; IV: independent variable; Coef: coefficient; SE: standard error; Pval: p-value; Cov: covariance; Med: mediation; Reg: regression.

## Data Availability

All data is presented in the text.
